# The effect of nanohydroxyapatite serum and toothpaste on prevention of enamel demineralization around orthodontic brackets: an in vitro study

**DOI:** 10.1186/s12903-025-07014-x

**Published:** 2025-10-14

**Authors:** Neda Babanouri, Farima Heidari, Fatemeh Hajipour, Hamidreza Pakshir

**Affiliations:** 1 Orthodontic Research Center, School of Dentistry, Shiraz University of Medical Science, Shiraz, Iran; 2 Postgraduate Student Dept. of Radiology, School of Dentistry, Shiraz University of Medical Sciences, Shiraz, Iran; 3 Postgraduate Student Dept. of Orthodontics, School of Dentistry, Shiraz University of Medical Sciences, Shiraz, Iran

**Keywords:** Nanohydroxyapatite, Dental enamel, Tooth demineralization, Orthodontic brackets

## Abstract

**Objectives:**

This prospective in vitro study aimed to evaluate the preventive effects of nanohydroxyapatite (nanoHAP), delivered as serum and toothpaste, in comparison with fluoride toothpaste, on enamel demineralization and surface microhardness around orthodontic brackets.

**Materials and methods:**

Forty-eight human enamel specimens were randomly allocated to three groups (*n* = 16). In Group 1, samples were brushed twice daily with fluoride toothpaste (1450 ppm) for 30 days, and nanoHAP serum (30% concentration) was applied during the final 10 days. Group 2 received only nanoHAP-containing toothpaste (10% concentration), while Group 3 was treated with fluoride toothpaste (1450 ppm) throughout the entire period. Orthodontic brackets were bonded to all specimens prior to a 14-day pH cycling regimen. Knoop microhardness was assessed on the buccal enamel surface at occlusal and cervical regions, 50 and 150 μm from the bracket margins, at depths of 10, 50, and 90 μm, without removing the brackets. DIAGNOdent was used for non-invasive enamel surface assessment before and after pH cycling. Data were analyzed using ANOVA and Duncan’s post hoc test (α = 0.05).

**Results:**

Significant differences were observed based on treatment material, positions, and their interactions (*P* < 0.05). Group 1 (nanoHAP serum + fluoride) showed significantly higher microhardness values and lower DIAGNOdent scores compared to Groups 2 and Group 3, indicating superior protection against demineralization.

**Conclusions:**

Short-term application of nanoHAP serum for 10 days, in combination with regular fluoride toothpaste, significantly reduces enamel demineralization around orthodontic brackets in vitro and may be a promising adjunctive strategy for preventing white spot lesions during orthodontic treatment.

## Introduction

Enamel demineralization, which clinically manifests as white spot lesions (WSLs), is characterized by a subsurface loss of minerals from the enamel, leading to increase porosity and a milky white opaque appearance on the tooth surface. These lesions, precursors to enamel caries, present both esthetic concerns for patients and functional challenges for clinicians, potentially impacting treatment outcomes and long-term oral health [1–3]. Consequently, developing effective preventive strategies is crucial in contemporary orthodontic practice [4–6]. A multitude of approaches have been explored to prevent enamel demineralization during orthodontic treatment or in general preventive contexts, including improved oral hygiene practices, fluoride-containing products, casein phosphopeptide–amorphous calcium phosphate (CPP-ACP), bioactive delivery systems such as fluoride-releasing adhesives, ozone therapy, and antimicrobial strategies such as probiotics and metal oxide nanoparticles (e.g., titanium dioxide, zinc oxide) [7–15].

Among these, nanohydroxyapatite (nanoHAP) has emerged as a promising biomimetic remineralizing agent due to its chemical and morphological similarity to the natural apatite crystals of enamel [16]. NanoHAP particles approximately 20 nm in size possess a higher surface-to-volume ratio and stronger enamel-binding potential compared to their larger or amorphous counterparts, enhancing their integration into enamel and facilitating remineralization [17, 18]. This allows the formation of a protective layer on the enamel surface, potentially enhancing resistance to subsequent acid challenges and reducing the risk of demineralization [17]. Previous studies have demonstrated the versatility of nanoHAP in various dental applications, such as implant coatings, sinus augmentation, dentin hypersensitivity treatment, enamel erosion prevention, and bone regeneration [19–24]. Its remineralizing effects on enamel and dentin, as well as its potential to prevent demineralization, have also been examined in both in vitro and in vivo models [25–31].

However, despite its demonstrated potential, few studies have investigated the prophylactic effect of nanoHAP specifically in the context of orthodontic treatment, particularly on bracketed enamel surfaces exposed to cariogenic challenges. Most existing research has focused on general enamel surfaces or various demineralization models, leaving a significant knowledge gap regarding targeted application of nanoHAP within the unique microenvironment created by orthodontic appliances.

Therefore, this in vitro study aimed to compare the effects of nanoHAP serum and nanoHAP-containing toothpaste—with a direct comparison between the two—and conventional fluoride toothpaste on enamel demineralization and surface microhardness around orthodontic brackets using Diagnodent and Knoop microhardness tests. The alternative hypothesis was that both nanoHAP products would reduce enamel demineralization more effectively than fluoride toothpaste, with nanoHAP serum expected to provide superior protective effects due to its higher concentration and delivery mechanism. These findings may have important clinical implications for the incorporation of nanoHAP-based products as complementary preventive agents in orthodontic care.

## Materials and methods

### Sample Preparation and surface treatment

Forty-eight sound human premolars extracted for orthodontic purposes were collected from the Department of Oral and Maxillofacial Surgery, School of Dentistry, Shiraz University of Medical Sciences. All extractions were performed as part of routine orthodontic treatment plans and not specifically for this research. Written informed consent was obtained from all patients, clearly stating that their extracted teeth could be used for research purposes. This study was approved by the Ethics Committee of Shiraz University of Medical Sciences, Shiraz, Iran (code: IR.SUMS.DENTAl.REC.1398.105). The study was performed in accordance with the Declaration of Helsinki developed by World Medical Association. Teeth with visible cracks, enamel hypoplasia, caries, restorations on the buccal surface, or prior chemical treatment (e.g., alcohol, formalin, hydrogen peroxide) were excluded. Extracted teeth were cleaned and stored in distilled water at room temperature, which was regularly replaced to prevent dehydration and maintain structural integrity. While distilled water lacks inherent antibacterial properties, its frequent replacement helps physically eliminate accumulating microorganisms, thereby minimizing bacterial colonization over the storage period [32, 33].

Sample size calculation was performed using G*Power version 3.1.9.2 (Faul, Erdfelder, Buchner, & Lang, 2014), based on the comparison of mean Diagnodent values among the three experimental groups. An effect size of 1.22, derived from a previous study [34], a significance level (α) of 0.05, and a power (1 − β) of 0.80 were used for the calculation. The analysis indicated that a minimum of 12 teeth per group (total *N* = 36) would be required. To further enhance the statistical power and account for potential variability, the sample size was increased to 16 teeth per group, resulting in a total of 48 specimens included in the study. The teeth were randomly assigned to three equal groups (*n* = 16) according to the treatment protocols: nanoHAP serum, nanoHAP toothpaste, and a control group. Each extracted tooth was first labeled with a unique identifier, and then a computer-generated random number sequence was used to allocate the teeth into one of the three groups. This process ensured an unbiased distribution of specimens across the experimental conditions. The baseline enamel condition was assessed via a laser fluorescence device (DIAGNOdent LF2190, KaVo, Biberach, Germany) with a “B” probe. For each tooth, three measurements were taken by two calibrated examiners across three days. Inter-examiner reliability was assessed before initiating the measurements, with the Kappa coefficient indicating an acceptable level of agreement (κ = 0.77, *P* < 0.001). The DIAGNOdent device was calibrated against a porcelain reference before every 5–6 measurements. The probe was held perpendicular to the tooth surface at the standardized distance from the future bonding area, and the maximum DIAGNOdent value was recorded. The mean of the three values was used as the baseline score (T0).

### Treatment protocols

Treatments were administered over a 30-day period as follows:

- Group 1 (nanoHAP Serum): Teeth were manually brushed twice daily for 20 s with a soft-bristled toothbrush and a standard toothpaste (Signal, Unilever France, Rueil-Malmaison Cedex, France) containing 1450 ppm fluoride. During the final 10 days, nanoHAP serum (PrevDent nanoHAP Repairing Serum, PrevDent International BV, Netherlands) containing 30% nanoHAP was applied to the buccal surface via the provided applicator for 2–3 min after evening brushing. After a 20-minute wait, the samples were rinsed with distilled water.
- Group 2 (nanoHAP Toothpaste): Teeth were brushed twice daily for 20 s with nanoHAP toothpaste (Signal Expert Protection, Unilever France, Rueil-Malmaison Cedex, France) containing 10% nanoHAP via a soft-bristled toothbrush.
- Group 3 (Control): Teeth were manually brushed twice daily for 20 s with a soft-bristled toothbrush and fluoride toothpaste (Signal, Unilever France, Rueil-Malmaison Cedex, France) containing 1450 ppm fluoride.

### Bracket bonding

Stainless steel orthodontic brackets (Mini Master Brackets, MBT prescription, 0.022-inch slot; American Orthodontics, USA) were bonded to the middle third of the buccal surfaces. The bonding protocol included (1) etching with 37% phosphoric acid gel (Reliance, Itasca, Illinois, USA) for 30 s; (2) application of a liquid primer (Transbond XT, 3 M Unitek, Monrovia, CA, USA) to the etched surface; and (3) placement of a thin layer of adhesive resin (Transbond XT, 3 M Unitek) on the bracket base. (4) Removal of excess adhesive and light curing for 40 s (10 s from each direction: mesial, distal, occlusal, and gingival).

A 2-mm margin of unbonded enamel surrounding each bracket was covered with acid-resistant nail varnish to limit the area exposed to demineralization during pH cycling.

### pH cycling protocol

To simulate cariogenic conditions, the samples were subjected to a 14-day pH cycling regimen. Each 24-hour cycle consisted of the following steps: (1) Demineralization phase: immersion in 60 mL of demineralizing solution (2.0 mmol/L calcium, 2.0 mmol/L phosphate, 75 mmol/L acetate, pH 4.3) for 6 h at 37 °C. (2) Remineralization phase: Rinsing with deionized water followed by immersion in 40 mL of remineralizing solution (1.5 mmol/L calcium, 0.9 mmol/L phosphate, 150 mmol/L potassium chloride, 20 mmol/L cacodylate buffer, pH 7.0) for 17 h at 37 °C [35].

After the cycling period, the teeth were removed, and DIAGNOdent readings were reassessed (T1) at four points around each bracket: cervical, occlusal, mesial, and distal.

Table 1 presents the DIAGNOdent values and the manufacturer’s recommended treatments.

**Table 1:** DIAGNOdent Pen Values and Recommended Clinical Action

DIAGNOdent Pen Values	No action	Preventive therapy	Record monitor	Sealant	Preparation
0–5	*				
5–10	*	*			
10–15	*	*	*	*	
15–20		*	*	*	
20–25		*	*	*	*
25–30		*	*	*	*
30+		*			*

### Cross-sectional microhardness analysis

**Fig. 1:**
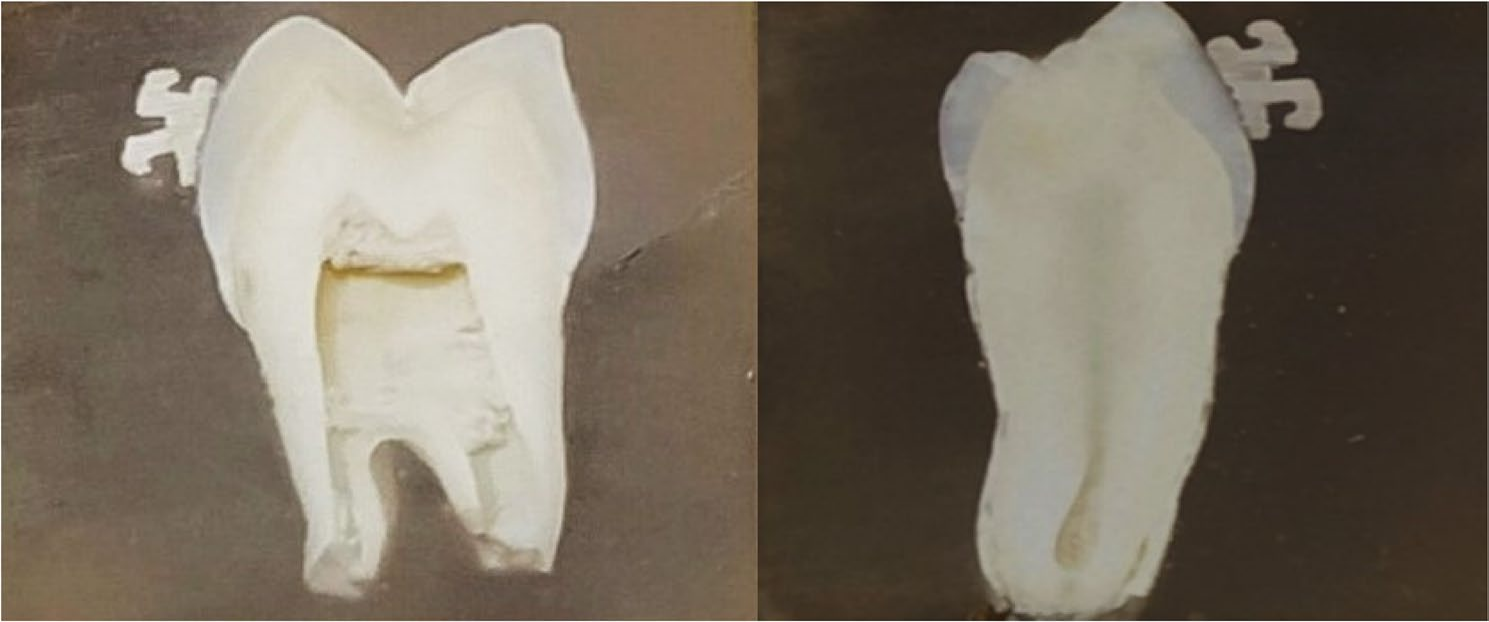
enamel specimens mounted in polyester resin block

Specimens were embedded in self-curing polyester resin (Sepahanrozitanrezin, Isfahan, Iran) to facilitate precise sectioning and microhardness evaluation. Brackets were not removed prior to sectioning to preserve the original bracket-enamel interface. Each specimen was sectioned buccolingually through the center of the bracket using a water-cooled, double-faced diamond disk (Crown cutter, Zircut, DFS Diamon, Germany) (Fig. 1). The exposed buccal enamel surfaces of the cross-sections were then polished with abrasive papers of increasing grit size (FlexiDisc, Cosmedent, Germany) to prepare them for hardness testing.

**Fig. 2:**
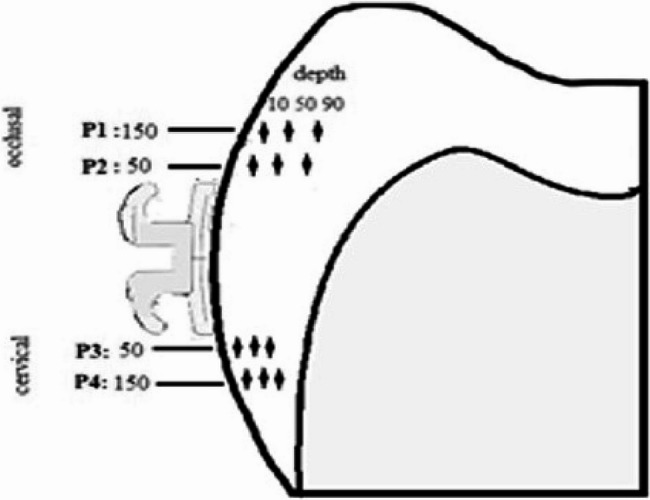
Diagrammatic representation of positions and depth of indentations

**Fig. 3:**
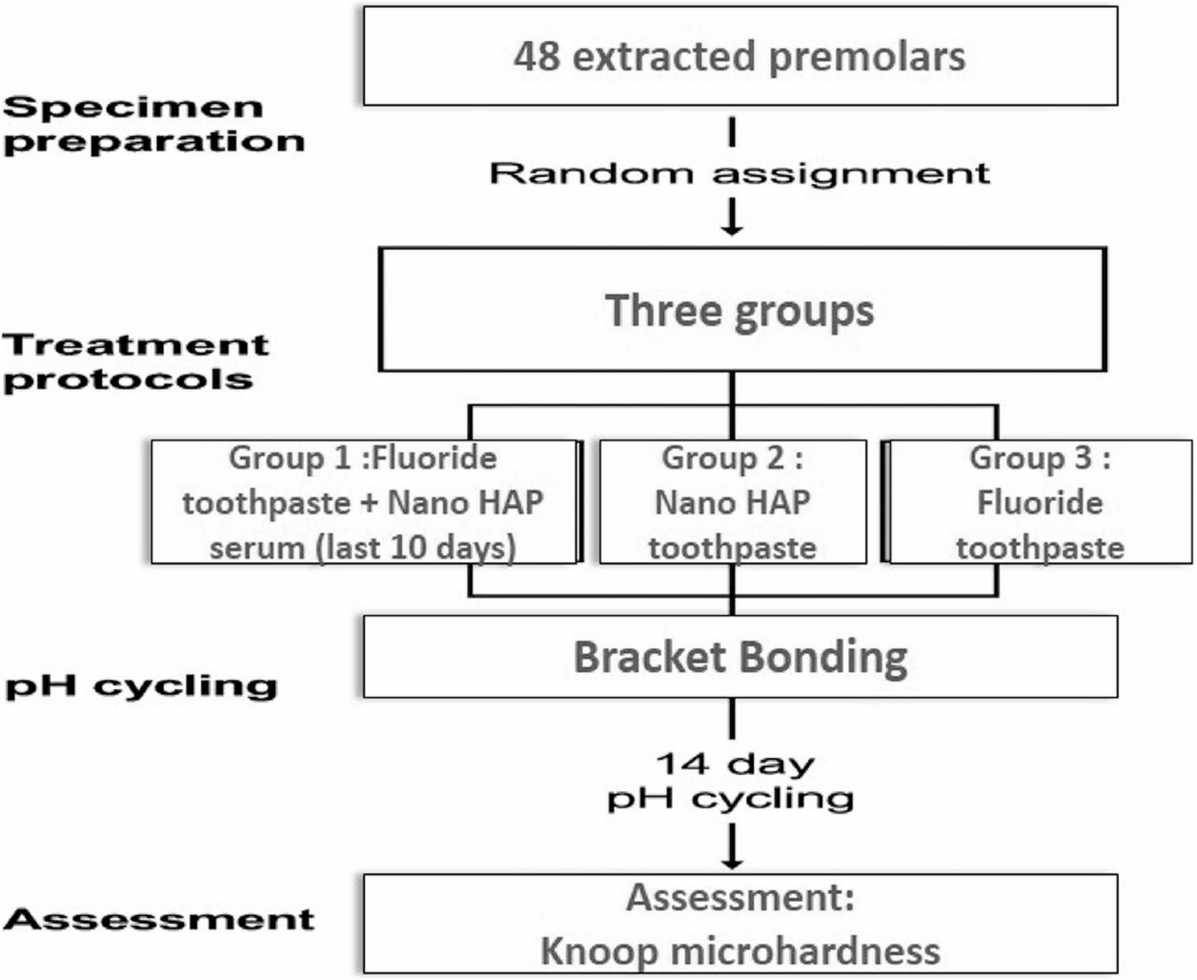
Schematic representation of the experimental design. Forty-eight premolars were randomly divided into three groups (nanoHAP serum, nanoHAP toothpaste, and fluoride toothpaste), treated for 30 days, bonded with brackets, and subjected to 14 days of pH cycling. Demineralization was assessed using DIAGNOdent readings and cross-sectional Knoop microhardness testing

A single blinded operator performed the microhardness analysis using a microhardness tester (MHV-1000Z, SCTMC, China) equipped with a Knoop indenter, applying a 50 g load for 5 s. Each half-crown received 12 indentations: three enamel depths (10, 50, and 90 μm from the surface) at four standardized locations (occlusal and cervical, 50 and 150 μm from the bracket) (Fig. 2). This cross-sectional indentation strategy allowed for a detailed assessment of the demineralization depth profile across treatment groups. Although baseline enamel hardness was not measured before intervention due to the destructive nature of sectioning, comparative post-treatment values after pH cycling allowed for evaluation of the relative effectiveness of each preventive protocol. A flowchart outlining the experimental design, including grouping, treatment protocols, pH cycling, and outcome assessment, is presented in Fig. 3.

### Statistical analysis

Data were analyzed using NCSS software (version 15.0, SPSS Inc., Chicago, Illinois, USA). A two-way ANOVA was used to evaluate the effects of treatment (nanoHAP serum, nanoHAP toothpaste, and fluoride toothpaste), depth from the enamel surface (10, 50, and 90 μm), position (occlusal and cervical, 50 and 150 μm from the bracket), and their interactions. Duncan’s post-hoc test was employed for multiple comparisons following a significant ANOVA results. Pairwise comparisons between group comparisons for DIAGNOdent scores and specific microhardness were performed using the Least Significant Difference (LSD) test. Paired t-tests were used to compare DIAGNOdent readings before and after treatment (T0 vs. T1) within each group. A significance level of *P* < 0.05 was considered statistically significant.

## Results

### Inter-examiner reliability for diagnodent measurements

The Kappa coefficient value indicated an acceptable level of inter-examiner agreement for Diagnodent (κ = 0.77, *P* < 0.001), indicating excellent agreement between the two examiners.

### Knoop microhardness analysis

Two-way ANOVA revealed significant effects of material type, position, and their interaction (material × position) on enamel microhardness (*P* < 0.001, Table 2). Depth analysis revealed similar trends: At 10, 50, and 90 μm, enamel treated with nanoHAP serum showed higher microhardness values than the other groups, with differences reaching statistical significance (*P* < 0.001, Table 5). However, depth alone, position × depth, and the three-way interaction (position × material × depth) were not statistically significant.

**Table 2:** ANOVA Results Assessing the Effects of Material Type, Measurement Position, and Depth on Enamel Knoop Microhardness

Source	Degrees of freedom	Sum of Squares	Ave. Square error	F	*P* (α)
Material	2	208682.623	104341.311	73.635	0.000*
Position	4	42128.866	10532.217	7.433	0.000*
Depth	2	3021.099	1510.549	1.066	0.345
Material/Position	8	57307.591	7163.449	5.055	0.000*
Material/Depth	4	1800.487	450.122	0.318	0.000*
Position/Depth	8	5663.411	707.926	0.500	0.857
Position/Material/Depth	16	8977.283	561.080	0.396	0.984

*Statistically significant (*P* < 0.05)

Analysis of the material × position interaction (Table 3) indicated that the nanoHAP serum group consistently presented the highest Knoop microhardness values at nearly all enamel positions. Significant differences between materials were observed at the 50 μm occlusal and cervical depths, as well as at the 150 μm cervical depth (*p* < 0.05). In the 150 μm occlusal region, the differences did not reach statistical significance (*p* = 0.098). Pairwise comparisons (Table 4) confirmed that nanoHAP serum showed significantly higher values than fluoride toothpaste at three of the four measured positions.

**Table 3:** Knoop microhardness (Avg ± SD) for materials and different positions

Interaction of Position/material	nanoHAP serum	nanoHAP toothpaste	fluoride toothpaste	*P* (α)
Occlusal/150 µm	194.9 ± 38.3	176.2 ± 36.9	162.8 ± 46.3	0.098
Occlusal/50 µm	195.2 ± 48.5	154.647 ± 31.3	139.1 ± 16.6	0.000*
Cervical/50 µm	176.9 ± 36.3	157.976 ± 18.4	137.5 ± 26.1	0.002*
Cervical/150 µm	190.7 ± 34.6	180.0 ± 29.2	141.2 ± 23.5	0.000*
P (α)	0.476	0.321	0.216	

*Differences between materials statistically significant by Duncan test

**Table 4:** Pairwise comparison between groups at different positions

		*P* (α)		
	P1	P2	P3	P4
nanoHAP serum vs. fluoride toothpaste	0.080	0.000*	0.003*	0.000*
nanoHAP tooth paste vs. fluoride toothpaste	0.222	0.457	0.160	0.087
nanoHAP serum vs. nanoHAP toothpaste	0.338	0.078	0.553	1.000

*Differences between materials statistically significant by LSD test

†P1: occlusal/150µm, P2: occlusal/50 µm, P3: cervical/50 µm, P4: cervical/150 µm

With respect to the material × depth interaction (Table 5), nanoHAP serum showed greater microhardness values than both the nanoHAP and fluoride toothpastes at all three depths (10 μm, 50 μm, and 90 μm; differences were statistically significant, *p* < 0.001). At 10 μm, no significant difference was observed between the nanoHAP and fluoride toothpaste groups. However, at 50 μm and 90 μm, the hardness of the nanoHAP toothpaste group was significantly greater than that of the fluoride group (Table 6). While the values in the nanoHAP toothpaste group were slightly greater than those in the fluoride group were, this difference was statistically significant only at 50 μm (*P* = 0.042) and 90 μm (*P* = 0.012) (Table 6).

**Table 5:** Knoop microhardnes (Avg ± SD) for materials at different depths from enamel surface

Interaction of Depth/material	nanoHAP serum	nanoHAP toothpaste	fluoride toothpaste	*P* (α)
10 μm	195.6 ± 23.1	163.3 ± 16.1	154.132 ± 20.0	0.000*
50 μm	193.8 ± 14.7	163.3 ± 18.2	149.412 ± 21.6	0.000*
90 μm	187.3 ± 20.9	163.2 ± 13.0	147.0 ± 16.5	0.000*
P (α)	0.540	0.868	0.587	

*Differences between materials statistically significant by Duncan test

**Table 6:** Pairwise comparison between groups at different depths

		*P* (α)	
	D0	D1	D2
nanoHAP serum vs. fluoride	0.000*	0.000*	0.000*
nanoHAP toothpaste vs. fluoride	0.165	0.042*	0.012*
nanoHAP serum vs. nanoHAP toothpaste	0.002*	0.006*	0.017*

*Differences between materials statistically significant by LSD test

†D_0_: 10 μm, D_1_: 50 μm, D_2_: 90 μm

### DIAGNOdent readings

The baseline DIANOGent scores (T0) were similar among all groups (*p* = 0.24), confirming comparable initial mineralization (Table 7). After pH cycling (T1), the DIAGNOdent values increased significantly in all the groups (*P* < 0.001), indicating demineralization. Following pH cycling (T1), all groups presented a significant increase in DIAGNOdent values, reflecting enamel demineralization (*p* < 0.001 for all groups). Posttreatment DIAGNOdent scores were lower in the nanoHAP serum group compared with the nanoHAP and fluoride toothpaste groups, with differences reaching statistical significance (*p* < 0.001, Table 8). No statistically significant difference was observed between the nanoHAP toothpaste and fluoride toothpaste groups (*p* = 0.07), although the former had a slightly lower mean value.

**Table 7:** Comparison of DIAGNOdent readings between T0 and T1 for each groups

groups	T0	T1	*P* (α)
nanoHAP serum	1.07 ± 0.15	5.26 ± 0.92	0.00*
nanoHAP tooth paste	0.93 ± 0.45	8.68 ± 0.74	0.00*
fluoride toothpaste	1.1 ± 0.63	9.26 ± 0.89	0.00*
P (α)	0.24	0.00*	

*Differences between T0-T1 statistically significant by pair T test

* Differences between materials statistically significant by LSD test

†T0: DD readings before pretreatment, T1: DD readings after pH- cycling

**Table 8:** Pairwise comparison between groups at T1

roups	*P* (α)
nanoHAP serum vs. nanoHAP tooth paste	0.00*
nanoHAP serum vs. fluoride toothpaste	0.00*
nanoHAP tooth paste vs. fluoride toothpaste	0.07

*Differences between materials statistically significant by LSD test

## Discussion

White spot lesions are a prevalent complication of fixed orthodontic treatment and are driven primarily by enamel demineralization adjacent to brackets. Although fluoride-based products remain the conventional standard for caries prevention, their limitations in subsurface remineralization have prompted the exploration of alternative strategies [36–38]. The present study evaluated the effects of nanoHAP in two delivery forms—serum and dentifrice—compared with fluoride toothpaste on enamel microhardness and mineral loss around orthodontic brackets under simulated cariogenic conditions. The results indicated that nanoHAP serum improved enamel microhardness and reduced demineralization compared with fluoride and nanoHAP dentifrices, with the most pronounced effects observed in our experimental model.

The extent of enamel demineralization was assessed via cross-sectional microhardness and laser fluorescence (DIAGNOdent), both of which are established and complementary analytical methods. Enamel hardness reflects its resistance to penetration by an indenter and serves as a key indicator of mineral content and structural integrity. Microhardness measurements using a reduced load (25–50 g) provide a sensitive and reliable means to detect changes in enamel strength due to demineralization and remineralization [39, 40]. Paschos et al. demonstrated a strong correlation between microhardness measurements and confocal laser scanning microscopy (CLSM) in evaluating enamel demineralization, indicating that both methods are effective for detecting subsurface lesions [40].

One of the findings of this study was the significant interaction effect between the treatment material and enamel position on the microhardness values. This finding indicates that the effectiveness of each agent varies depending on the specific location on the enamel surface. In particular, nanoHAP serum resulted in significantly higher microhardness values in the cervical regions, which are more susceptible to plaque accumulation and demineralization. This localized effect may be related to the formulation characteristics of the serum, which could allow better interaction with enamel prisms, particularly in regions with irregular surface morphology. Considering the increased susceptibility of cervical enamel to demineralization, the enhanced performance of nanoHAP serum in this region supports its potential as a targeted preventive strategy for high-risk areas during orthodontic treatment.

In addition to location, the significant interaction between material and depth reveals another clinically relevant dimension. NanoHAP particles, due to their nanoscale size (~ 20 nm) and high surface area, mimic the natural hydroxyapatite crystals of enamel, enabling them to penetrate enamel microporosities and subsurface lesions more effectively than fluoride compounds. This biomimetic property may support integration into the enamel structure, potentially promoting remineralization beneath the surface layers, which is important as early carious lesions often begin sub-surface where the effect of fluoride is limited. In contrast, fluoride primarily promotes the formation of fluorapatite on the enamel surface, reinforcing only the outermost layer without substantial penetration. This difference in penetration capability explains the significant depth-dependent increase in microhardness observed with nanoHAP serum at depths of 10, 50, and 90 μm in our study.

Interestingly, nanoHAP toothpaste—despite containing the same active ingredient—consistently underperformed compared with the serum, especially in deeper regions. This discrepancy may be related to the lower nanoparticle concentration and shorter application time of the toothpaste during brushing, emphasizing the importance of formulation characteristics and delivery methods in determining clinical efficacy [41].

The clinical utility of DIAGNOdent in detecting incipient enamel lesions has also been demonstrated in earlier investigations, particularly in pediatric populations, where early diagnosis is essential to guide preventive strategies [42, 43]. Previous studies have reported a strong correlation (*r* = 0.91) between enamel microhardness and the mineral content of carious lesions [44]. The DIAGNOdent results revealed significantly lower mineral loss in the nanoHAP serum group than in both the nanoHAP and fluoride toothpastes, indicating better preservation of enamel integrity following acid etching and pH cycling. These findings are in agreement with the microhardness data, further confirming the superior protective effect of the serum. This enhanced efficacy may be attributed to its higher concentration and purity of active components, which facilitate the formation of a more stable mineral layer on the enamel surface. The longer contact time and reduced presence of abrasive or interfering agents in the serum compared with toothpaste formulations may further support its interaction with enamel, potentially enhancing protective performance under cariogenic conditions.

Investigations have demonstrated the beneficial role of nanoHAP in promoting enamel remineralization, minimizing demineralization, and preventing the formation of white spot lesions during orthodontic treatment [27, 45–47]. Comparative studies further revealed the efficacy of nanoHAP over other remineralizing agents. For example, de Carvalho et al. [26] reported superior protective effects of nanoHAP in preventing enamel demineralization compared with those of CCP-ACP paste and fluoride varnish in an in vitro model. An in situ study by Souza et al. [29] revealed that a 10% nanoHAP and 0.2% fluoride formulation (Nanop Plus) was more effective than MI paste in enhancing enamel remineralization and reducing dentin demineralization. These results align with the findings of the current study, which also support the superior efficacy of nanoHAP in preventing enamel demineralization. These findings support the potential of nanoHAP as an effective agent for enhancing enamel protection.

Although the protective effects of nanoHAP have been explored, the majority of studies have focused on its therapeutic application following the onset of enamel demineralization rather than its preventive potential when applied prior to bracket bonding [48–51]. However, studies specifically addressing the preventive effects of nanoHAP prior to bracket bonding and before the onset of enamel demineralization remain limited. Among the few available studies, one in vivo study reported reduced demineralization with combined nanoHAP and fluoride use, but the lack of separate groups made it unclear whether the effect was due to the combination or individual components [52]. The present study addresses this limitation by adopting a structured experimental design that includes separate groups for nanoHAP, fluoride and their combination. The potential of nanoHAP as a preventive agent prior to bracket bonding is important, as it can increase enamel resistance before demineralization begins, reduce the impact of acid etching, and offer early protection during the critical initial phase of orthodontic treatment.

The clinical feasibility of incorporating nanoHAP into orthodontic protocols depends on its compatibility with the bonding process. A previous study investigated the effect of nanoHAP application on the shear bond strength of brackets and reported no adverse impact, even when various bonding approaches, such as sandblasting and altered etching durations, were applied [53]. This finding is particularly relevant, as it suggests that nanoHAP can be safely used prior to bracket bonding without compromising the mechanical retention of orthodontic appliances. Therefore, considering both its demonstrated preventive efficacy and its neutral effect on shear bond strength, nanoHAP has emerged as a promising adjunct for enhancing enamel protection in orthodontic patients, especially during the early stages of treatment when the risk of demineralization is high.

This study shows the clinical potential of nanoHAP as a preventive agent in orthodontics. By enhancing enamel resistance before bracket bonding, nanoHAP helps reduce the risk of white spot lesions during the early stages of treatment when patients are adjusting to new oral hygiene protocols. Its effectiveness, together with its negligible impact on bond strength, may support nanoHAP as a safe and promising adjunct for enamel protection in high-risk orthodontic patients.

This study has several limitations that should be considered when interpreting the results. The in vitro design, although useful for maintaining controlled conditions, does not fully replicate the complex oral environment, including factors such as saliva, dietary influences, oral hygiene, and patient cooperation, and masticatory or physiological forces [35]. The pH-cycling protocol used to simulate acidic challenges did not involve direct measurement of pH changes or buffering capacity, which may affect the reproducibility and interpretation of the demineralization process. The experimental model also lacked a biofilm or microbiological component, which plays a crucial role in the development of enamel demineralization under clinical conditions. The study focused on short-term acid exposures and did not assess the long-term durability of the protective effects of nanoHAP under repeated acid challenges that may occur during orthodontic treatment. Baseline enamel microhardness could not be measured due to the destructive nature of cross-sectional sectioning. Only two indirect assessment methods (DIAGNOdent and microhardness testing) were employed; additional analyses using scanning electron microscopy (SEM), energy-dispersive X-ray spectroscopy (EDX), or micro-computed tomography (micro-CT) would provide a more comprehensive evaluation of morphological and mineral changes in enamel. Manual brushing may introduce variability in treatment application, and using only a relatively small number of premolar teeth may limit generalizability. The absence of an untreated control group limited the ability to establish a baseline for comparing enamel microhardness across the experimental groups. These methodological constraints should be considered when interpreting the findings and planning future clinical studies. Awareness of these limitations is important when applying the findings and planning future clinical studies, which should incorporate complementary analyses (SEM, EDX, or micro-CT) and evaluate the long-term benefits, patient compliance, and cost-effectiveness of nanoHAP serum compared with conventional fluoride regimens in vivo.

## Conclusion

Within the limitations of this in vitro investigation, the 10-day application of nano-hydroxyapatite (nanoHAP) serum prior to orthodontic bracket bonding, in conjunction with regular fluoride toothpaste use, showed higher microhardness values and lower DIAGNOdent scores around brackets compared with the use of fluoride or nanoHAP toothpaste alone. These findings suggest that nanoHAP serum may serve as an adjunctive pretreatment for enhancing enamel resistance against demineralization in patients undergoing fixed orthodontic therapy. Further clinical studies are needed to evaluate these outcomes under in vivo conditions.

## Data Availability

The datasets used and analyzed during the current study are available from the corresponding author on reasonable request.

## Abbreviations

nanoHAP: Nanohydroxyapatite
WSLs: White Spot Lesions
CPP-ACP: Casein Phosphopeptide-amorphous Calcium Phosphate
LSD: Least Significant Difference
CLSM: Confocal Laser Scanning Microscopy
SEM: Scanning Electron Microscopy
EDX: Energy-Dispersive X-ray Spectroscopy
micro-CT: Micro-computed Tomography
